# Announcement

**DOI:** 10.1155/S1064744995000767

**Published:** 1995


					AN ANNOUNCEMENT FROM THE
INFECTIOUS DISEASES SOCIETY
FOR OBSTETRICS AND GYNECOLOGY
The Infectious Diseases Society for Obstetrics and Gynecology (IDSOG) was created in 1973 to
gatherprofessionals inthe fieldofobstetrics andgynecologyinterestedinthe scientific studyofinfectious
disease.
Any qualified physician or biologist who is a member or an associate ofa department ofobstetrics
andgynecologywithinateachinginstitutionis eligible fornominationto annualmembership. Prospective
members mustdemonstrate interest inthe studyofinfectious diseasesby 1)havinghadaminimum offive
articles related to infectious diseases published in peer reviewedjournals, ofwhich the candidate is the
primaryauthorofatleastthree; 2)presentingatleastone scientificpaperatthe annualmeetingone ormore
years prior to being eligible for membership; and 3) be sponsored and seconded by members in good
standing ofthe Society. Annual dues for the Society are $75. There are currently 78 members.
The Society's annual scientific meeting and symposium will be held August 4-17, 1996 at the Hyatt
Regency Beaver Creek in Beaver Creek, CO. A symposium on "Premature Labor" will be held on
Wednesday, August 14 from 1:00-5:00 p.m. Thursday through Saturday includes presentation of
abstracts.
For furtherinformationwrite to: Jane Jenkins, IDSOG, 409 12th Street SW, Washington DC 20024-
2188 or call (202)863-1648, fax (202)554-0453 or e-mail jjenkins@acog.com.
262 INFECTIOUS DISEASES IN OBSTETRICSAND GYNECOLOGY

				

